# One Year Facing COVID. Systematic Evaluation of Risk Factors Associated With Mental Distress Among Hospital Workers in Italy

**DOI:** 10.3389/fpsyt.2022.834753

**Published:** 2022-03-10

**Authors:** Matteo Bonzini, Anna Comotti, Alice Fattori, Filippo Cantù, Elisa Colombo, Valentina Tombola, Eralda Myslymi, Michele Gatti, Giulia Stucchi, Carlo Nava, Lorenzo Bordini, Luciano Riboldi, Paolo Brambilla

**Affiliations:** ^1^Occupational Medicine Unit, IRCCS Maggiore Policlinico Hospital Foundation, Milan, Italy; ^2^School of Medicine, University of Milan, Milan, Italy; ^3^Psychiatric Unit, IRCCS Maggiore Policlinico Hospital Foundation, Milan, Italy

**Keywords:** healthcare workers, mental health, risk factors, psychological impairment, COVID-19 vaccine

## Abstract

**Introduction:**

Italy was the first Western country affected by the COVID-19 pandemic that still constitutes a severe challenge for healthcare workers (HCWs), with a deep impact on their mental health. Several studies confirmed that a considerable proportion of HCW developed adverse psychological impairment (PsI). To focus on preventive and rehabilitation measures, it is fundamental to identify individual and occupational risk factors. We systematically assessed possible PsI among all employees in a large university hospital in Italy, using validated psychometric scales in the context of occupational health surveillance.

**Methods:**

In the period of July 2020 to July 2021, we enrolled 990 HCWs. For each subject, the psychological wellbeing was screened in two steps. The first-level questionnaire collected gender, age, occupational role, personal and occupational COVID-19 exposure, general psychological discomfort (GHQ-12), post-traumatic stress symptoms (IES-R), and anxiety (GAD-7). Workers showing PsI (i.e., test scores above the cutoff in at least one among GHQ-12, IES-R, and GAD-7) have been further investigated by the second-level questionnaire (psycho-diagnostic) composed by PHQ-9, DES-II, and SCL-90 scales. If the second-level showed clinically relevant symptoms, then we offered individual specialist treatment (third level).

**Results:**

Three hundred sixteen workers (32%) presented signs of PsI at the first-level screening questionnaire. Women, nurses, and subjects engaged in the COVID-19 area and with an infected family member showed significantly higher PsI risk. PsI prevalence was strongly associated with the pandemic trend in the region but sensibly decreased after January 2021, when almost all workers received the vaccination. A proportion of subjects with PsI presented clinically relevant symptoms (second-level screening) on PHQ-9 (35%), DES (20%), and SCL-90 (28%). These symptoms were associated neither to direct working experience with patients with COVID-19 nor to COVID-19 experience in the family and seemed not to be influenced by the pandemic waves or workers vaccination.

**Conclusions:**

The evaluation of psychological wellbeing of all hospital workers, directly or indirectly exposed to pandemic consequences, constitutes a unique condition to detect individual, occupational, and non-occupational risk factors for PsI in situations of high stress and/or disasters, as well as variables associated with symptom chronicization.

## Background and Aims

Italy was the first Western country to be affected by the COVID-19 pandemic since February 2020, when the exponential rise of cases required a national lockdown and imposed a rapidly increasing extraordinary amount of work on the healthcare system in terms of critical care and reorganization.

Under such circumstances, healthcare workers (HCWs) experienced heavy workload, physical exhaustion, frustration and helplessness, and fear of infecting themselves and their relatives (1). Thus, besides physical safety, HCWs' mental health was a major concern for authorities (2) and occupational physician. Moreover, studies conducted during previous epidemics [SARS, MERS, and Ebola; (3, 4)] and primary studies conducted in China at the very beginning of the COVID-19 pandemic showed a high prevalence of post-traumatic stress disorder (PTSD), depression, and anxiety disorders among HCWs (5–7). More recently, several studies, including reviews, have been conducted from the very beginning of the COVID-19 pandemic on HCW mental health and confirmed that a considerable proportion of workers developed adverse psychological outcomes during the COVID pandemic (8–12). These studies found that being frontline workers, female gender, younger age, lower job seniority, and nursing profession predicted worsened mental health (13, 14).

Most of these studies are focused on critical care workers and data collected through web-based questionnaires, being able in several cases to collect only a proportion of the workers' data. Thus, results could be partially affected by the self-selection of respondents, and the comparison of mental health outcomes between more exposed workers and other colleagues is limited. Another relevant common limitation is the lack of information of non-occupational important risk factors (such as COVID infection in the family): although HCWs of intensive care units faced a large number of COVID-19 deaths and substantial work-related stress, all healthcare professionals were also exposed to personal grief and family concerns (15). Finally, because the majority of the published studies were conducted during the first phases of the pandemic, results are focused on the early onset symptoms with little evidence on the persistence of symptoms and delayed-onset PTSD, which typically occurs a few months after exposure.

This is why we point out that, even in the current pandemic scenario, it is crucial to evaluate and monitor the mental health of HCWs during different phases and waves of the COVID pandemic (1) to prevent possible mental disorders, (2) to discover work-related and individual risk factors that can exacerbate psychological distress, and (3) to target rehabilitation strategies on more vulnerable people. For these reasons, we designed a prospective study that systematically evaluates the mental wellbeing of all workers employed in a large second-level general hospital in Milan, Italy. They were followed by the occupational physician health surveillance, using a multistep approach to assess psychological workload and symptoms with validated scales. The study covered almost a period of 1 year and has been characterized by two waves of the epidemic as well as a massive and rapid campaign of health workers' vaccination that in our region (Lombardy) occurred in January to February 2021.

## Methods

### Study Design

We developed a multi-step process to evaluate workers mental health to encourage participation with a first brief screening and then offering further support to those who need it. To take into account the requirements of both brevity and validity, we adopted extensively used screening instruments for common psychological impairment (PsI) related to COVID-19 pandemic [an extensive description of the methodology adopted for this study was illustrated in a previous report (16)]. We proposed our screening to all workers employed in our hospital.

1. First level: to detect possible PsI with standardized scales during a structured medical-assisted interview in the context of occupational health surveillance;
2. Second level: when first-level scales show PsI, workers are invited to undergo a second-level questionnaire to better assess possible psychological distress;
3. Third level: to offer a specialist evaluation and psychological support and/or psychiatric treatment to workers who show specific symptoms at the second-level questionnaire.

We plan to perform a follow-up re-evaluation on all participants within 12 months from enrollment to evaluate trends in psychological burden, recognize delayed onset of symptoms, and evaluate the efficacy of specialist treatments.

### Setting and Participants

The study is conducted jointly by the units of Occupational Medicine and Psychiatry.

From July 2020 onward, all workers have been invited to participate, independently from age, sex, department, and job title. The only two exclusion criteria were being employed after the beginning of the study and the refusal to sign the informed consent; there were no exclusion criteria on pre-existing pathologies, aiming to include the overall and most general pool of the population. An extended informed-consent form has to be signed before the first-level evaluation. Formal ethical approval was also obtained from the Hospital Ethical Committee in July 2020.

### Assessment Measures

First-level evaluation is composed of an occupational physician interview collecting (i) socio-demographic characteristics (age and gender); (ii) occupational data, including information about occupational role (administrative staff, heath assistant, nursing staff, physicians, and others), hospital unit/department, and engagement in COVID-19 area (none, concluded, and still ongoing) with respective intensity (high/low) and length; and (iii) clinical information regarding chronic conditions and habitual medications, specifying which drugs were taken after pandemic began and a psychometric questionnaire.

The questionnaire is collected directly on digital support and consists of the following:

- The General Health Questionnaire (GHQ-12) (17) in the validated Italian version (18, 19) for assessing psychological distress and short-term changes in mental health. We adopted the dichotomous scoring method (0-0-1-1) and a score above or equal to 4 as the cutoff point (20, 21).
- Impact of Event Scale-Revised (IES-r) for assessing post-traumatic stress symptoms (22). A brief description guides subjects to answer the following questions by assessing their subjective responses related to the COVID-19 emergency in the previous 7 days with 22 questions exploring intrusion, avoidance, and hyperarousal symptoms. A total score of 33 on the IES-r yielded a diagnostic sensitivity of 0.91 and specificity of 0.82 (23). The Italian version has also shown optimal psychometric properties and validity (24).
- Generalized Anxiety Disorders (GAD-7) (25) to screen anxiety symptoms. With robust psychometric properties and strong validity, a score of 10 or greater represents a reasonable cutoff point to identify cases of GAD; increasing scores on the GAD-7 are also strongly associated with multiple domains of functional impairment and disability.
- A section collecting individual COVID-19 exposure and COVID-related health concerns/beliefs: to have been positive of COVID-19 and duration of the condition, to have been in quarantine and duration, to have family members that tested positive/were hospitalized/died of COVID-19, personal concern for infecting family members, the experience of social discrimination outside the hospital, changes in family's habits, thoughts about changing job, fear for their own safety, and the experience of moral injury at work.

The second-level questionnaire contains specific scales to further investigate psychopathological symptoms and disorders:

- Symptom Checklist-90-Revised (SCL-90-R) (26) is a self-administered scale for the evaluation of psychiatric symptomatology;
- The Dissociative Experience Scale II (DES II) (27, 28). Dissociative symptoms are frequently found in the aftermath of trauma and occur to some degree in individuals without mental disorders and are thought to be more prevalent in persons with major mental illnesses. The DES II has been developed to offer a means of reliably measuring dissociation in normal and clinical populations;
- Patient Health Questionnaire-9 (PHQ-9) (29). The PHQ-9 is aimed at assessing depression disorder by scoring each of the nine DSM-IV criteria.

A specialist psychiatric feedback of second-level evaluation results is sent to the occupational physician who, if tests are indicative of impairment in psychological functioning, proposes to the worker a specialist consultation in person. That third-level evaluation is comprised of the specialist consultation within 1 week from the second-level evaluation and is followed, according to every single case, by an eventual psychiatric follow-up or psychotherapy. To individuate late signs and to assess individual changes in psychological distress, all subjects repeat tests after no more than 12 months.

### Statistical Analysis

Data were collected through an automatic database generated by the REDCap platform (30), which was subsequently analyzed by R software (31). An independent coded dataset accessible only to the PI guarantees data protection linking individual information (i.e., name and surname) with an alphanumeric code.

Statistical analysis was aimed to individuate risk factors for sub-optimal psychological wellbeing and/or impaired psychological function.

In univariate analysis, the relationship between each potential risk factor and outcomes, treated as continuous variables, was preliminarily investigated in terms of mean differences across subgroups through independent samples *t*-test and one-way ANOVA. Comparison in the percentage of subjects with a total score higher than the cutoff for each scale was evaluated through the Chi-square test.

In multivariate analysis, each potential risk factor is included in multiple logistic regression models to explore the relative contributions [in terms of odds ratios (ORs)] of the various risk factors to the dependent variables including potential covariates and confounders. The overall significance of each variable was tested through the likelihood ratio test.

The relationship between personal concerns and feelings about COVID-19, collected through six questions with multiple answers (not at all, little, enough, and very), and first-level outcome variables was graphically explored, and the difference in the distribution was investigated through the Kolmogorov–Smirnov test for discrete variables. To study their effect on first-level scores in terms of risk factors, they have been converted into dichotomous variables (yes = not at all and little; no = enough and very) and put one by one in the multivariate logistic regression model.

The effect of vaccination on psychological scales has been investigated exploring differences between workers enrolled before and after the COVID-19 vaccination campaign, which started in January 2020. To study how the effect of risk factors, in particular of the variables related to COVID-19 exposure, varied after the vaccination, we performed multivariate logistic regression on first-level screening dividing the dataset into two sub-samples (*N* = 584 and *N* = 406, before and after vaccination campaign, respectively). The significance of the relationship between these variables and vaccination was evaluated including an interaction term in the multivariate logistic regression model on the whole dataset, using a binary variable indicating enrollment before or after the vaccination campaign.

A *p* < 0.05 will be considered statistically significant. ORs are calculated with their relative 95% confidence intervals.

## Results

The occupational medicine unit, where workers underwent the periodical health surveillance already prescribed by the current Italian legislation, proposed the study protocol to all workers since July 2020. By July 2021, we had enrolled 990 subjects out of a total population of 1,610. The participation rate was 62%. In detail, 220 (13%) workers did not answer our calls or were unavailable and 400 (25%) refused to participate.

Table 1 summarizes the numbers and main characteristics of enrolled subjects and the results of the first-level questionnaires. The percentage of subjects scoring above the cutoff of the first-level scales widely differed by gender, age, occupational role, and COVID-19 exposure at work and in their own family. No significant differences were found dividing subjects with or without a previous COVID-19 infection (stated by a positive swab). Similar results were found considering average values in each psychometric scale, instead of cutoffs.

**Table 1:** First level screening scales across subgroups: number of enrolled subjects, means, standard deviations and frequencies of scorings above the cutoff at the different first level psychometric scales.

		**GHQ-12**		**IES-R**		**GAD-7**	
	***N* (%)**	**Mean (sd)**	***N* (%) > cutoff**	**Mean (sd)**	***N* (%) > cutoff**	**Mean (sd)**	***N* (%) > cutoff**
**Gender**
Male	297 (30%)	2.79 (3.07)	96 (32%)	16.2 (15.3)	46 (16%)	4.58 (4.43)	44 (15%)
Female	693 (70%)	3.27 (3.32)	270 (39%)	20.5 (17.0)	146 (21%)	6.38 (5.30)	161 (23%)
*p*-value		0.03*	0.06***	<0.001*	0.05***	<0.001*	0.003***
**Age group**
20–30	137 (14%)	3.73 (3.54)	62 (45%)	20.6 (16.5)	30 (22%)	6.55 (4.93)	33 (24%)
30–40	276 (28%)	3.21 (3.17)	110 (40%)	19.3 (15.5)	55 (20%)	5.92 (4.84)	56 (20%)
40–50	245 (24.5%)	3.27 (3.43)	90 (37%)	19.9 (18.6)	53 (22%)	6.13 (5.60)	60 (25%)
>50	332 (33.5%)	2.72 (3.02)	104 (31%)	17.9 (16.0)	54 (16%)	5.27 (5.02)	56 (17%)
*p*-value		0.01**	0.02***	0.35**	0.32***	0.06**	0.17***
**Occupational role**
Administrative staff	119 (12%)	2.44 (2.83)	34 (29%)	16.8 (14.3)	14 (12%)	5.32 (4.92)	20 (17%)
Health assistant	63 (6.5%)	2.67 (3.45)	17 (27%)	23.1 (18.2)	15 (24%)	5.98 (5.23)	17 (27%)
Nursing staff	416 (42%)	3.79 (3.52)	188 (45%)	23.0 (18.4)	115 (28%)	6.71 (5.52)	111 (27%)
Physician	233 (23.5%)	2.81 (2.89)	80 (34%)	15.0 (13.6)	27 (12%)	4.96 (4.49)	34 (15%)
Others	159 (16%)	2.55 (2.97)	47 (29%)	15.6 (14.0)	21 (13%)	5.20 (4.68)	23 (14%)
*p*-value		<0.001**	<0.001***	<0.001**	<0.001***	<0.001**	<0.001***
**COVID-19 area working experience**
Never	544 (55%)	2.54 (2.92)	160 (29%)	16.7 (14.3)	72 (13%)	5.27 (4.79)	90 (17%)
Yes^†^
Previously	202 (20%)	3.63 (3.47)	86 (43%)	21.5 (17.9)	48 (24%)	6.04 (5.25)	46 (23%)
Currently	244 (25%)	4.01 (3.52)	120 (49%)	23.9 (18.6)	72 (30%)	7.04 (5.49)	69 (28%)
*p*-value		<0.001**	<0.001***	<0.001**	<0.001***	<0.001**	<0.001***
<4 months	227 (23%)	3.93 (3.54)	107 (47%)	22.7 (18.6)	58 (26%)	6.38 (5.44)	54 (24%)
>4 months	219 (22%)	3.74 (3.45)	99 (45%)	23.1 (18.1)	62 (28%)	6.81 (5.38)	61 (28%)
*p*-value		<0.001**	<0.001***	<0.001**	<0.001***	<0.001**	<0.001***
Low-intensity area	101 (10%)	3.26 (3.41)	37 (37%)	18.6 (15.2)	19 (19%)	5.70 (4.94)	21 (21%)
High-intensity area	345 (35%)	4.01 (3.51)	169 (49%)	24.1 (19.0)	101 (29%)	6.85 (5.52)	94 (27%)
*p*-value		<0.001**	<0.001***	<0.001**	<0.001***	<0.001**	<0.001***
**Positive nasoph. swab**
Yes	153 (15%)	3.15 (3.40)	55 (36%)	18.9 (16.2)	31 (20%)	5.89 (4.84)	28 (18%)
No	837 (85%)	3.13 (3.23)	311 (37%)	19.3 (16.7)	161 (19%)	5.83 (5.17)	177 (21%)
*p*-value		0.93*	0.83***	0.83*	0.85***	0.87*	0.48***
**Family member positive to COVID-19**
Yes	209 (21%)	3.43 (3.15)	89 (43%)	19.1 (15.6)	44 (21%)	6.04 (4.72)	45 (22%)
No	781 (79%)	3.16 (3.29)	277 (36%)	19.3 (16.9)	148 (19%)	5.79 (5.22)	160 (21%)
*p*-value		0.30*	0.07***	0.86*	0.56***	0.55*	0.9***

*t-test.

**One-way ANOVA.

***Chi-square test.

† *p-values refer to comparisons between subjects with working experiences in COVID-19 area (current/previous, number of days, intensity area) and subjects with no experience in COVID-19 area*.

Table 2 presents multivariate logistic regression analysis for first-level screening scales. Adjusted OR showed that gender, occupational role, working experience with patients with COVID-19, and having a family member with previous COVID-19 infection were risk factors for PsI. Women had an increased risk of developing anxiety symptoms by around 70% (see GAD-7 scale), being a nurse almost tripled the risk for developing symptoms of post-traumatic distress (see IES-R scale), almost doubled the risk of anxiety (GAD-7), and increased by 41% the risk of general discomfort (GHQ-12). Direct experience with patients with COVID-19 was associated with an increased risk of PsI in all three scales. In detail, the risk to score above the cutoff (for all measured scales) increased with time spent in the COVID-19 area, with a higher level of clinical intensity, or dividing subject with none, former, or current involvement in COVID-19 units.

**Table 2:** Multivariate logistic regression for f first level screening scales: adjusted OR for scoring above the cut-offs with associated 95% confidence intervals and corresponding LR test *p*-values.

		**GHQ-12**	**IES-R**	**GAD-7**
	***N* (%)**	**AdjOR (95% CI)**	**AdjOR (95% CI)**	**AdjOR (95% CI)**
**Gender**
Male	297 (30%)	1.00	1.00	1.00
Female	693 (70%)	1.37 (1.01, 1.85)	1.44 (0.99, 2.13)	1.72 (1.19, 2.54)
*p*-value		*0.04*	*0.06*	*0.003*
**Age**
>50	332 (33.5%)	1.00	1.00	1.00
20–30	137 (14%)	1.12 (0.72, 1.76)	0.69 (0.39, 1.20)	1.02 (0.59, 1.72)
30–40	276 (28%)	1.05 (0.73, 1.51)	0.79 (0.50, 1.24)	0.96 (0.61, 1.49)
40–50	245 (24.5%)	1.05 (0.73, 1.46)	1.06 (0.68, 1.66)	1.35 (0.88, 2.07)
*p*-value		*0.03*	*0.31*	*0.17*
**Occupational role**
Physician	233 (23.5%)	1.00	1.00	1.00
Administrative staff	119 (12%)	1.07 (0.63, 1.80)	1.58 (0.74, 3.27)	1.44 (0.75, 2.75)
Health assistant	63 (6.5%)	0.66 (0.34, 1.22)	2.27 (1.09, 4.61)	2.07 (1.04, 4.05)
Nursing staff	416 (42%)	1.41 (1.00, 2.01)	2.90 (1.82, 4.73)	1.95 (1.26, 3.06)
Others	159 (16%)	0.99 (0.62, 1.56)	1.60 (0.84, 3.05)	1.14 (0.75, 2.75)
*p*-value		*0.003*	* <0.001*	*0.007*
**COVID-19 area working experience**
Never	544 (55%)	1.00	1.00	1.00
Yes^†^
Previously	202 (20%)	1.75 (1.20, 2.52)	2.08 (1.31, 3.29)	1.43 (0.91, 2.22)
Currently	244 (25%)	2.27 (1.59, 3.25)	2.80 (1.82, 4.34)	1.96 (1.29, 2.96)
*p*-value		* <0.001*	* <0.001*	*0.007*
<4 months	227 (23%)	2.07 (1.44, 2.97)	2.26 (1.45, 3.54)	1.49 (0.97, 2.29)
>4 months	219 (22%)	1.95 (1.35, 2.82)	2.66 (1.71, 4.15)	1.93 (1.26, 2.96)
*p*-value		* <0.001*	* <0.001*	*0.009*
Low-intensity area	101 (10%)	1.41 (0.87, 2.28)	1.67 (0.90, 3.03)	1.35 (0.75, 2.37)
High-intensity area	345 (35%)	2.22 (1.61, 3.09)	2.69 (1.81, 4.05)	1.80 (1.23, 2.66)
*p*-value		* <0.001*	* <0.001*	*0.009*
**Positive nasoph. swab**
No	837 (85%)	1.00	1.00	1.00
Yes	153 (15%)	0.78 (0.53, 1.15)	0.94 (0.58, 1.48)	0.73 (0.45, 1.16)
*p*-value		*0.55*	*0.98*	*0.21*
**Family member positive**
No	781 (79%)	1.00	1.00	1.00
Yes	209 (21%)	1.48 (1.05, 2.08)	1.17 (0.77, 1.76)	1.11 (0.74, 1.65)
*p*-value		*0.02*	*0.64*	*0.61*

† *p-values refer to comparisons between subjects with working experiences in COVID-19 area (current/previous, number of days, intensity area) and subjects with no experience in COVID-19 area*.

For subjects with a family member that was previously infected by COVID-19, *the risk of general discomfort (GHQ-12) was increased by 48%*; age was not found as a significant risk factor for PsI.

Table 3 shows the univariate analysis for the second-level scales, collected among 316 subjects. Similar to first-level screening, gender and occupational role resulted as statistically significant factors associated with psychological distress: means and percentage of scoring above the cutoff were higher for females, nurses, and health assistants (although the latter are composed by a few cases). Contrary to first-level outcomes, working exposure to COVID-19 and having a family member with previous COVID infection were not associated with higher psychological scales scoring.

**Table 3:** Second level screening scales (*N* = 316): means, standard deviations and frequencies of scorings above the cutoff across subgroups.

		**PHQ-9**		**DES**		**SCL-90**	
	***N* (%)**	**Mean (sd)**	***N* (%) > cutoff**	**Mean (sd)**	***N* (%) > cutoff**	**Mean (sd)**	***N* (%) > cutoff**
**Gender**
Male	81 (26%)	8.63 (4.79)	22 (27%)	9.94 (10.4)	12 (15%)	0.66 (0.48)	17 (21%)
Female	235 (74%)	9.54 (5.44)	88 (37%)	13.2 (13.7)	50 (21%)	0.84 (0.64)	73 (31%)
*p*-value		0.16*	0.12***	0.03*	0.27***	0.01*	0.12***
**Age group**
20–30	57 (18%)	9.11 (5.15)	15 (26%)	11.3 (9.29)	9 (16%)	0.73 (0.57)	13 (23%)
30–40	91 (29%)	8.95 (5.29)	27 (30%)	14.4 (14.2)	27 (30%)	0.78 (0.61)	29 (32%)
40–50	81 (25.5%)	9.98 (5.49)	34 (42%)	11.3 (13.1)	13 (16%)	0.84 (0.60)	25 (31%)
>50	87 (27.5%)	9.18 (5.21)	34 (39%)	11.9 (13.8)	13 (15%)	0.81 (0.63)	23 (26%)
*p*-value		0.59**	0.14***	0.37**	0.04***	0.72**	0.62***
**Occupational role**
Administrative staff	27 (8%)	8.44 (5.01)	9 (33%)	14.4 (17.5)	6 (22%)	0.86 (0.71)	10 (38%)
Health assistant	16 (5%)	12.2 (4.62)	11 (69%)	21.3 (19.1)	8 (50%)	1.27 (0.83)	9 (56%)
Nursing staff	173 (55%)	10.3 (5.43)	64 (37%)	14.1 (13.3)	43 (25%)	0.86 (0.60)	55 (32%)
Physician	62 (20%)	7.34 (4.58)	13 (21%)	6.48 (6.29)	2 (3%)	0.54 (0.32)	6 (10%)
Others	38 (12%)	7.18 (4.46)	13 (34%)	8.92 (9.17)	3 (8%)	0.67 (0.61)	10 (26%)
*p*-value		<0.001**	0.008***	<0.001**	<0.001***	<0.001**	<0.001***
**COVID-19 area working experience**
Never	138 (44%)	8.65 (4.92)	47 (34%)	12.2 (12.8)	23 (17%)	0.80 (0.62)	41 (30%)
Yes^†^
Previously	64 (20%)	10.1 (5.47)	26 (41%)	12.0 (11.9)	16 (25%)	0.77 (0.60)	15 (24%)
Currently	114 (36%)	9.64 (5.56)	37 (32%)	12.8 (13.9)	23 (20%)	0.80 (0.59)	34 (30%)
*p*-value		0.13**	0.53***	0.89**	0.37***	0.95**	0.63***
<4 months	82 (26%)	10.0 (5.50)	31 (38%)	13.5 (14.1)	22 (27%)	0.77 (0.60)	19 (23%)
>4 months	96 (30%)	9.65 (5.55)	21 (33%)	11.6 (12.5)	17 (18%)	0.81 (0.58)	30 (31%)
*p*-value		0.14**	0.79***	0.61**	0.15***	0.93**	0.47***
Low-intensity area	30 (9%)	9.20 (5.46)	10 (33%)	12.9 (14.2)	8 (27%)	0.80 (0.69)	9 (30%)
High-intensity area	148 (47%)	9.94 (5.54)	53 (36%)	12.4 (13.1)	31 (21%)	0.79 (0.57)	40 (27%)
*p*-value		0.12**	0.93***	0.95**	0.39***	0.97**	0.86***
**Positive nasopharyngeal swab**
Yes	51 (16%)	9.69 (5.08)	18 (35%)	13.4 (15.7)	8 (16%)	0.76 (0.56)	12 (24%)
No	265 (84%)	9.23 (5.33)	92 (35%)	12.1 (12.5)	54 (20%)	0.80 (0.61)	78 (29%)
*p*-value		0.56*	0.9***	0.58*	0.56***	0.63*	0.47***
**Family member positive to COVID-19**
Yes	76 (24%)	9.14 (4.72)	23 (30%)	10.9 (9.91)	11 (15%)	0.74 (0.51)	16 (21%)
No	240 (76%)	9.36 (5.46)	87 (36%)	12.8 (13.9)	51 (22%)	0.81 (0.63)	74 (31%)
*p*-value		0.74*	0.41***	0.19*	0.25***	0.32*	0.12***

*t-test.

**One-way ANOVA.

***Chi-square test.

† *p-values refer to comparisons between subjects with working experiences in COVID-19 area (current/previous, number of days, intensity area) and subjects with no experience in COVID-19 area*.

Table 4 presents multivariate logistic regression analysis for psychological distress (second-level questionnaire results). Nurses and health assistants had sensibly higher adjusted OR for developing symptoms of depression or other psychological symptoms than physicians. ORs were greater in women considering all the three scales (even if not statistically significant). Similar to univariate analysis, the occupational exposure with COVID-19 seemed not to be an independent risk factor for psychological distress.

**Table 4:** Multivariate logistic regression for second level scales: adjusted OR of scoring above the cut-offs with associated 95% confidence intervals (CI) and corresponding LR test *p*-values.

		**PHQ-9**	**DES**	**SCL-90**
	***N* (%)**	**AdjOR (95% CI)**	**AdjOR (95% CI)**	**AdjOR (95% CI)**
**Gender**
Male	81 (26%)	1.00	1.00	1.00
Female	235 (74%)	1.40 (0.77, 2.60)	1.68 (0.80, 3.79)	1.48 (0.78, 2.94)
*p*-value		*0.14*	*0.1*	*0.11*
**Age**
>50	87 (27.5%)	1.00	1.00	1.00
20–30	57 (18%)	0.39 (0.17, 0.88)	0.78 (0.27, 2.21)	0.68 (0.328 1.64)
30–40	91 (29%)	0.44 (0.21, 0.91)	1.71 (0.72, 4.17)	1.05 (0.50, 2.24)
40–50	81 (25.5%)	0.93 (0.48, 1.79)	0.88 (0.35, 2.20)	1.19 (0.58, 2.48)
*p*-value		*0.14*	*0.05*	*0.61*
**Occupational role**
Physician	62 (20%)	1.00	1.00	1.00
Administrative staff	27 (8%)	2.12 (0.70, 6.40)	8.23 (1.61, 62.65)	5.41 (1.62, 19.6)
Health assistant	16 (5%)	9.45 (2.79, 36.3)	26.7 (5.48, 202.3)	11.9 (3.29, 47.5)
Nursing staff	173 (55%)	2.79 (1.34, 6.10)	8.53 (2.39, 54.6)	4.81 (1.99, 13.6)
Others	38 (12%)	2.35 (0.89, 6.30)	2.53 (0.39, 20.5)	3.52 (1.13, 11.8)
*p*-value		*0.004*	* <0.001*	* <0.001*
**COVID-19 area working experience**
Never	138 (44%)	1.00	1.00	1.00
Yes^†^
Previously	64 (20%)	1.59 (0.79, 3.20)	1.41 (0.60, 3.29)	0.71 (0.32, 1.51)
Currently	114 (36%)	1.32 (0.70, 2.50)	1.19 (0.54, 2.62)	1.20 (0.62, 2.33)
*p*-value		*0.34*	*0.65*	*0.38*
<4 months	82 (26%)	1.51 (0.78, 2.96)	1.55 (0.69, 3.48)	0.71 (0.34, 1.46)
>4 months	96 (30%)	1.35 (0.70, 2.60)	1.05 (0.46, 2.39)	1.27 (0.65, 2.49)
*p*-value		*0.38*	*0.44*	*0.27*
Low-intensity area	30 (9%)	1.19 (0.44, 3.09)	1.80 (0.57, 5.55)	1.03 (0.37, 2.75)
High-intensity area	148 (47%)	1.47 (0.82, 2.67)	1.20 (0.58, 2.52)	0.97 (0.52, 1.81)
*p*-value		*0.37*	*0.55*	*0.97*
**Positive nasopharyngeal swab**
No	51 (16%)	1.00	1.00	1.00
Yes	265 (84%)	0.92 (0.44, 1.88)	0.85 (0.32, 2.02)	0.80 (0.36, 1.71)
*p*-value		*0.6*	*0.52*	*0.31*
**Family member positive to COVID-19**
No	76 (24%)	1.00	1.00	1.00
Yes	240 (76%)	0.77 (0.41, 1.42)	0.66 (0.29, 1.41)	0.61 (0.30, 1.17)
*p*-value		*0.4*	*0.29*	*0.13*

† *p-values refer to comparisons between subjects with working experiences in COVID-19 area (current/previous, number of days, intensity area) and subjects with no experience in COVID-19 area*.

**Figure 1:**
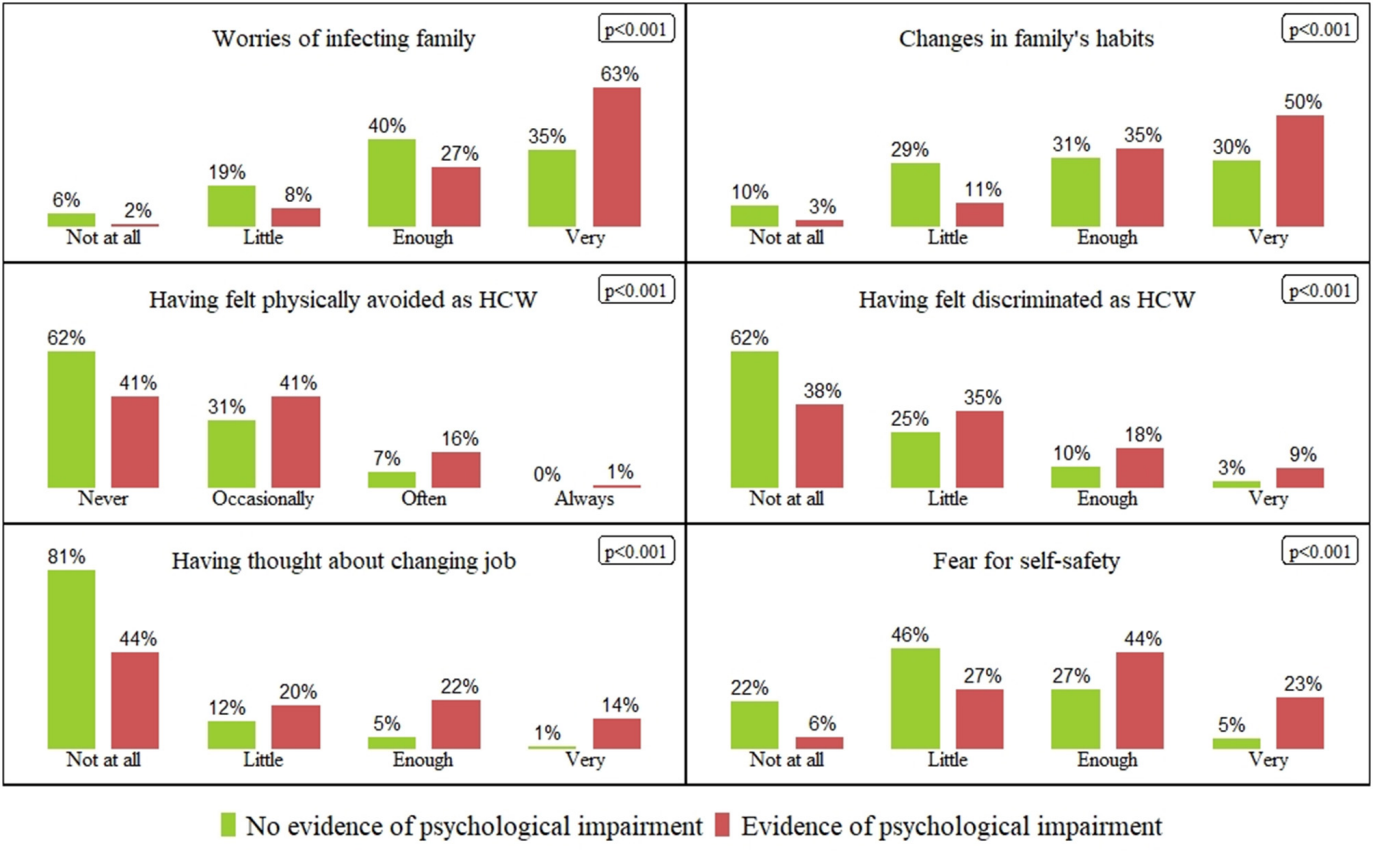
Health beliefs and COVID-19 related concerns: percentage of each answer dividing subjects with evidence of psychological impairment (red columns) and without psychological impairment (green columns).

Figure 1 illustrates the distribution of health beliefs and COVID-19 concerns for each answer, which significantly differed according to the first-level screening result (Kolmogorov–Smirnov test). Worries, discomfort, and fear were expressed more frequently by subjects who scored above the cutoff on at least one scale compared to colleagues with no evidence of PsI. Adjusted ORs of having a first-level scale above the cutoff dividing subjects according to their personal concerns and beliefs about COVID-19 are presented in Table 5. Each variable resulted in a statistically significant risk factor with a high OR, indicating a strong relationship with psychological distress. The highest risks that increased by more than six times were associated with thoughts about changing jobs and fear for self-safety.

**Table 5:** Personal concerns about COVID-19 and risk to score above the cut-off at the first levels scales (reference subject answering No).

		**GHQ-12**	**IES-R**	**GAD-7**
	***N* of positive (%)**	AdjOR*(95% CI)	AdjOR*(95% CI)	AdjOR*(95% CI)
Worries of infecting family	792 (80%)	2.43 (1.60, 3.47)	4.13 (2.30, 8.11)	2.15 (1.34, 3.59)
Changes in family's habits	695 (70%)	3.22 (2.31, 4.54)	4.89 (3.04, 8.25)	4.34 (2.78, 7.04)
Having felt physically avoided as HCW	111 (11%)	1.72 (1.13, 2.61)	3.50 (2.25, 5.43)	2.54 (1.63, 3.91)
Having felt discriminated as HCWs	179 (18%)	2.07 (1.44, 2.86)	3.46 (2.37, 5.03)	2.16 (1.48, 3.13)
Having thought about changing job	175 (18%)	6.71 (4.58, 10.0)	6.17 (4.21, 9.08)	6.38 (4.36, 9.37)
Fear for self-safety	445 (45%)	3.59 (2.72, 4.77)	5.65 (3.89, 8.35)	3.92 (2.79, 5.56)

*ORs are adjusted by gender, age group, occupational role, COVID-19 area, personal infection and family member infection.

**Figure 2:**
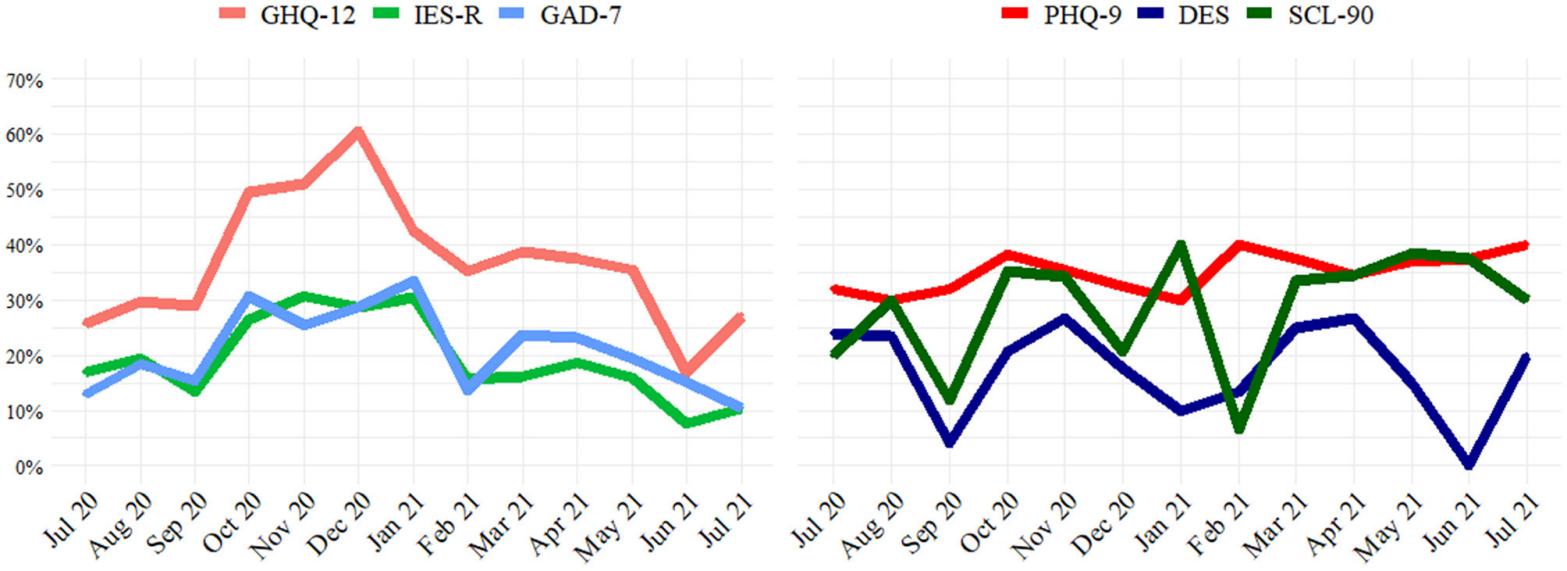
Time trend of first level screening **(left)** and second level evaluation **(right)**. Percentage of subjects scoring above scales cut-off over time.

Figure 2 shows the time trends in the percentage of subjects, resulting in scores above cutoff in first- and second-level scales. Looking at the first-level screening, the highest levels were reached between October and December 2020, during the second pandemic wave in Italy. In particular, the percentage above the cutoff of the GHQ-12 scale increased from September to December, reaching a peak of around 60%. A rapid increase in September to October was present also for GAD-7 and IES-R scales. From January 2021 percentages of subjects with PsI started to decrease, returning to baseline values in a few months.

Time trends of second-level questionnaires were more irregular and different from each other: percentage of overpass PHQ-9 cutoff was constant around 30–40%, and for DES and SCL-90, no clear trend during the study period was found.

In the period of January-February 2021, more than 90% of HCWs received anti–COVID-19 vaccination. We explored the effect of vaccination on psychological wellbeing, comparing results in subjects evaluated before and after the vaccination campaign started.

Values of OR for PsI related to exposure to the COVID-19 working area did not vary with vaccination: although statistical significance was lost in the post-vaccine subsample, results showed a stable increased risk among subjects working in the COVID-19 area. Similarly, a personal COVID-19 infection was not a risk factor before or after vaccination. Having a family member previously infected was a risk factor for PsI only for workers enrolled before the vaccination campaign (ORs are equal to 2.25 for GHQ-12, 1.46. for IES-R, and 1.71 for GAD-7) but not for vaccinated workers (ORs are equal to 1.18, 1.10, and 0.86, respectively). Detailed data for GHQ-12, IES-R, and GAD-7 scales are illustrated in Table 6.

**Table 6:** ORs (adjusted for gender, age, occupational role) of scoring above cut-off of first level screening scales before and after vaccination campaign.

	***N*** **(%)**		**GHQ-12**			**IES-R**			**GAD-7**		
	**PRE**	**POST**	**AdjOR (95% CI)**		***p***	**AdjOR (95% CI)**		***p***	**AdjOR (95% CI)**		***p***
	***N* = 584**	***N* = 406**	**PRE**	**POST**		**PRE**	**POST**		**PRE**	**POST**	
**COVID-19 area working experience**
Never		295 (73%)									
Yes^†^	249 (43%)		1.00	1.00		1.00	1.00		1.00	1.00	
Previously	133 (23%)	69 (17%)	1.72 (1.05, 2.83)	1.54 (0.82, 2.87)	0.75	1.97 (1.08, 3.64)	1.60 (0.72, 3.49)	0.67	1.44 (0.79, 2.63)	1.15 (0.54, 2.38)	0.71
Currently	202 (34%)	42 (10%)	1.99 (1.25, 3.18)	2.64 (1.23, 5.70)	0.71	2.25 (1.29, 4.01)	2.55 (0.94, 6.63)	0.73	1.86 (1.07, 3.28)	1.66 (0.66, 4.01)	0.64
Low-intensity area	65 (11%)	36 (9%)	1.11 (0.59, 2.06)	2.04 (0.87, 4.76)	0.3	1.19 (0.52, 2.59)	2.30 (0.77, 6.61)	0.56	1.31 (0.61, 2.72)	0.99 (0.33, 2.72)	0.71
High-intensity area	270 (46%)	75 (18%)	2.17 (1.40, 3.39)	1.80 (0.99, 3.27)	0.57	2.47 (1.45, 4.31)	1.74 (0.80, 3.69)	0.68	1.78 (1.05, 3.07)	1.43 (0.70, 2.85)	0.58
**Positive nasoph. swab**
No	515 (88%)	322 (79%)	1.00	1.00		1.00	1.00		1.00	1.00	
Yes	69 (12%)	84 (21%)	0.58 (0.32, 1.03)	1.03 (0.60, 1.77)	0.28	1.00 (0.51, 1.88)	1.00 (0.47, 2.01)	0.84	0.52 (0.24, 1.04)	0.98 (0.49, 1.85)	0.58
**Family member positive to COVID-19**
No	500 (86%)	281 (69%)	1	1		1	1		1	1	
Yes	84 (14%)	125 (31%)	2.25 (1.34, 3.83)	1.18 (0.73, 1.91)	0.06	1.46 (0.81, 2.58)	1.10 (0.57, 2.05)	0.28	1.71 (0.95, 3.03)	0.86 (0.47, 1.54)	0.11

*P-values are referred to the significance of the interaction term*.

† *p-values refer to comparisons between subjects with working experiences in COVID-19 area (current/previous, number of days, intensity area) and subjects with no experience in COVID-19 area*.

## Conclusions

We conducted a 12-month-long systematic evaluation of mental health in all workers that underwent occupational surveillance (*n* = 990) in a tertiary hospital in Milan that was identified as one of the COVID-19 hub centers in the Lombardia Region (Italy). Our study investigated psychological wellbeing (by GAD-7, IES-R, and GHQ-12) and specific psychiatric symptoms (by PHQ-9, DES, and SCL-90) with a focus on risk factors associated with mental health issues.

As consistently stated by the previous investigation, PsI was more frequent among nurses and female workers (13, 14, 32).

By comparing psychological scales in workers with or without direct involvement with patients with COVID, we observed a statistically increased risk for impairments (in all considered scales) in exposed workers, which was confirmed when we considered the duration of employment in COVID wards (>6 months, <6 months, and none) and the level of intensity of care (high, low, and none). This is consistent with research on previous coronavirus outbreaks, showing the exposure level as a major risk factor for mental health problems (9, 33). On the other hand, we observed a not negligible proportion of workers with PsI even in HCWs without experience with patients with COVID-19 and among administrative staff (34). These results are both compatible with a background proportion of mental health issues in the working population and with the effect of pandemic-related changes and concerns that involved the entire working population. COVID-19 pandemic represented a psychological challenge and a trigger of psychological distress for all, and our data confirmed that personal concerns and health beliefs related to COVID-19 (e.g., worries about infection or about infecting family members) strongly impact the risk for PsIs.

In this regard, our observation of increased psychological distress in workers as having a family member with previous COVID-19 infection confirmed the multidimensional (occupational and non-occupational) impact of the pandemic on workers' mental health (35, 36).

Three hundred and sixteen workers (32%) presented signs of PsI at the first-level screening (i.e., with scores above the cutoff in at least one scale among GAD-7, IES-r, and GHQ-12); among these, only a proportion of subjects presented clinically relevant symptoms (second-level screening) on PHQ-9 (35%), DES (20%), and SCL-90 (28%). The relative frequency of PsI was strongly associated with the pandemic trends in the region (with a rapid increase in the last trimester 2020) but sensibly decreased after January 21, when almost all workers received the vaccination. Differently, specific psychiatric symptoms showed a different pattern of association with potential risk factors and different time trends compared to PsI. In fact, results of second-level scales were associated neither to direct working experience with patients with COVID nor to COVID experience in the family and seemed not to be influenced by pandemic waves or workers vaccination. Instead pre-existing and more stable conditions (specifically gender and occupational levels) resulted associated with sensibly higher ORs.

These results are not completely surprising as psychiatric symptoms may have pre-existed and therefore are not associated with COVID-19 risk factors; also, we cannot exclude that a self-selection bias had occurred as HCW involved in high-intensity wards may have more resilience, psychological wellbeing and better coping resources compared to colleagues involved in other wards (37, 38).

However, to detect susceptible populations that develop psychiatric problems in a context of generalized and persistent stress, as was the experience during the pandemic, it is a key challenge in terms of occupational medicine. For example, the higher proportion of mental health issues observed among nurses and health assistants (when compared with doctors) is a matter of concern and suggests targeting specific efforts and care to preserve psychological wellbeing in those working groups.

Our results must be considered in light of several limitations. First of all, we have no data collected before COVID. Thus, we cannot attribute to the pandemic, all the observed psychological distress. We were aware that psychological symptoms are present in all working populations and that HCWs, in particular, experienced a high level of job stress and even burnout from work shifts, long working hours, and several other job-related psychological risk factors. However, the increasing trend in PsIs with increasing direct working involvements with patients with COVID suggested that care for patients with COVID had a specific and independent effect in determining psychological burden even if (or maybe because of) HCWs constitute a population previously exposed to a high level of job strain.

We collected both exposure and effect with questionnaires; thus, our study is prone to potential biases as self-selection of respondents (39) and common methods bias (40). We managed to minimize those risks grounding our investigation on the occupational physician health surveillance (obtaining a very high participation rate and minimizing the risk of untrue or uncompleted answers in describing job tasks) and by assessing individual “COVID exposure” by objective data (hospital wards, duration of employments, and swab results etcetera).

Our results about the effect of vaccination campaigns among HCWs are interesting and, nowadays, represent one of the first shreds of evidence collected in Europe. However, we were not able to evaluate each worker before and after vaccination, and we only compared mental wellbeing in the same population in the period before and after the vaccination campaign. Thus, we cannot exclude that the better psychological scores observed were a consequence of another unmeasured time-dependent factor, first of all, a general improvement of the pandemic situation in Italy. In this respect, we must say that, in Italy, vaccination among HCWs was performed sensibly before (2–4 months as average) the general population, and we experienced, within the study period (March to July 2021), a sensible increase of cases and hospital admission (COVID-19 pandemic third wave in Europe) without observing an evident effect on workers psychological burden after their vaccination.

Our study plans to follow all enrolled workers for another year to properly assess both late onsets of symptoms, to analyze the risk factors for symptoms persistency, and to overcome some of the abovementioned limitations. The next results may provide further insights on preventive and beneficial interventions to support HCW mental health during and after a pandemic. Indeed, different programs aimed at addressing mental health issues in HCWs during pandemics have been found to be effective (41, 42). In this respect, it is also crucial to maintain an ongoing cooperation with public health stakeholders, policymakers, and the occupational health and safety players within hospital contexts (43).

The evaluation of the psychological wellbeing of all hospital workers, directly or indirectly exposed to pandemic consequences, constitutes a unique condition to detect individual, occupational, and non-occupational risk factors for PsI in situations of high stress and/or disasters, as well as variables associated with symptom chronicization.

## Data Availability Statement

The datasets presented in this article are not readily available because collected variables include information about occupational stress, job satisfaction and psychological wellbeing. For ethical reasons, to avoid any possible workers identification, data are available only in aggregate format upon reasonable request to the corresponding authors. Requests to access the datasets should be directed to matteo.bonzini@unimi.it; paolo.brambilla@unimi.it.

## Ethics Statement

The studies involving human participants were reviewed and approved by Milan Area 2 Ethical Committee, n.652_2020 of July 21, 2020. The patients/participants provided their written informed consent to participate in this study.

## Author Contributions

MB, AF, and PB conceived the study, wrote the study protocol, and wrote the paper. MB and AC performed the statistical analyses. FC, EC, and VT supervised the second level questionnaires and contributed to study interpretation. EM, MG, LB, CN, and GS supervised the first level questionnaire and performed occupational health surveillance. LR supervised the study and contributed to results interpretation. All authors contributed to the article and approved the submitted version.

## Funding

The full cost of the study was covered by the Fondazione IRCCS Ca' Granda Ospedale Maggiore Policlinico as the study was carried out within the occupational health surveillance contest required by the Italian regulation on occupational health and safety (i.e., Legislative Decree n.81/2008).

## Conflict of Interest

The authors declare that the research was conducted in the absence of any commercial or financial relationships that could be construed as a potential conflict of interest.

## Publisher's Note

All claims expressed in this article are solely those of the authors and do not necessarily represent those of their affiliated organizations, or those of the publisher, the editors and the reviewers. Any product that may be evaluated in this article, or claim that may be made by its manufacturer, is not guaranteed or endorsed by the publisher.
